# Effect of health rights accessibility on the urban integration of minority rural migrants in China: a cross-sectional study

**DOI:** 10.1186/s12889-024-18294-3

**Published:** 2024-03-11

**Authors:** Qingjun Zhao, Guosong Wu, Hanrui Wang

**Affiliations:** 1 College of Economics and Management, Huzhou College, Huzhou, China; 2 School of Economics and Management, Huzhou University, Huzhou, China; 3 Institute of Sustainable Development, Huzhou University, Huzhou, China; 4 College of Economics and Management, Nanjing Agricultural University, Nanjing, China

**Keywords:** Health rights accessibility, Urban integration, Minority rural migrants, China, Health integration

## Abstract

**Background:**

Accessing health rights is an integral component of people’s aspirations for a better life. Existing discussions and evaluations regarding the accessibility of health rights for minority rural migrants are insufficient. In comparison to objective health conditions, inequalities in health rights lead to chronic and long-term depletion of human capital among minority rural migrants. This study aimed to assess the overall impact, heterogeneity effects, and mechanisms of health rights accessibility on the urban integration of minority rural migrants.

**Methods:**

Based on the 2017 China Migrants Dynamic Survey Data (CMDS), this study employs OLS models, 2SLS models, conditional mixed process (CMP) methods, and omitted variable tests to estimate the impact of health rights accessibility on the urban integration of minority rural migrants. Additionally, from the perspectives of migration scope and illness experience, this study explored the heterogeneity in the relationship between health rights accessibility and urban integration. Finally, using the Karlson–Holm–Breen (KHB) model, this study dissects the mechanisms through which health rights accessibility influences the urban integration of minority rural migrants.

**Results:**

Health rights accessibility significantly enhances the urban integration of minority rural migrants. Moreover, compared to minority rural migrants who move across provinces and who have no history of illness, those who migrate within the same province and who have experienced illness are more sensitive to the positive impact of health rights accessibility. However, the enhancing effect of health rights accessibility does not significantly differ between the new and old generations of minority rural migrants. Furthermore, health rights accessibility can indirectly improve the urban integration of minority rural migrants by elevating health levels, improving health habits, and reinforcing health behaviors. Among these, the indirect effects mediated by health habits are more pronounced.

**Conclusion:**

The research conclusions underscore the issue of health accessibility and urban integration among minority rural migrants, providing a reexamination and clarification of the policy effects of health rights in promoting the urban integration of minority rural migrants. Relevant policy design should commence with improving the health rights of minority rural migrants, enhancing their health integration capabilities, and effectively boosting their ability to integrate into urban life.

**Supplementary Information:**

The online version contains supplementary material available at 10.1186/s12889-024-18294-3.

## Background

With the continuous acceleration of China’s economic and social development, coupled with the rapid urbanization process, the scale and growth rate of minority migration have been expanding. Issues such as quality improvement, health security, and urban integration have drawn increasing amounts of attention. According to data from the National Bureau of Statistics of China, in 2020, the total number of minority migrants nationwide reached 33.71 million, an increase of 25.47 million compared to the year 2000, representing a staggering growth of 309.1%. Due to the risks associated with frequent mobility, the vulnerability of migrant employment, the challenges of urban integration, and the continuity of social security, minority migrants are prone to health risks [1–3]. Previous studies have found that the quantity and quality of public health services provided to rural migrants are insufficient, leading to serious concerns about their health conditions [4–7]. Compared to urban residents, rural migrant populations are not only more vulnerable to infectious diseases but also more likely to become potential spreaders. It is worth noting that unlike other countries, China has implemented a household registration system since 1958, dividing household registration into agricultural and non-agricultural categories based on geographical and family relationships [8]. This system links social welfare benefits such as children’s education, employment services, social security, healthcare, and housing to household registration status. As a result, the national basic public health service system is divided into two relatively independent subsystems for urban and rural areas, and rural migrants can only access services and exercise rights through the rural basic medical and health service system [9–11]. This means that rural migrants can only enjoy the same rights to public health services as residents after obtaining household registration in their destination cities. In order to improve the accessibility of public health services for rural migrants, enhance their health literacy, and help this group integrate into urban life more effectively [12]. In recent years, the Chinese government has implemented a series of public health service policies for the floating population, gradually providing equal public health services for rural migrants. In 2013, the National Health Commission of China initiated a pilot project for equalizing public health services for the floating population. By 2016, the Central Committee of the Communist Party of China and the State Council issued the “Healthy China 2030 Planning Outline.” Subsequently, in the 19th Party Congress report, the emphasis was on accelerating the equalization of basic public services, establishing a high-quality and efficient medical and health service system. All these policies underscored the importance of public health services for rural migrants, aiming to enhance the “sense of access” for the floating population by providing equalized public health services. Therefore, studying the accessibility and universality of public health rights for minority migrants is crucial.

A significant portion of minority rural migrants relocate from their hometowns to urban areas in pursuit of better job opportunities, higher income, and the aspiration to become urban residents [13]. Existing studies indicate that the essence of the urbanization process can be understood not only as the transformation of rural and urban household registrations but also as the gradual process by which rural migrants gradually enjoy the same basic public services as local urban residents [14, 15]. However, it is crucial to note that minority rural migrants can only enjoy equal public health services as local residents after completing household registration migration [13, 16]. In comparison to the nation’s high attention and rapid progress in the top-level design and practical implementation of public health services, there is a somewhat lagging development in standardized and in-depth academic research in the field of public health services. Research has focused mainly on various aspects of the implementation progress, equalization levels, factors influencing the equalization of public health services, and impacts generated by public health services. In terms of practical outcomes, there is still a certain gap between the awareness and utilization of health rights, including minority rural migrants, and the goals of the basic equalization policy for public health services [12, 17, 18]. Studies based on different survey data consistently confirm that upon arrival, minority rural migrants not only face challenges such as poor living conditions and weak health awareness [19, 20] but also relatively low proportions of establishing health records and receiving health education [21, 22]. Accessibility to medical facilities is inferior to that of the registered population [14], resulting in lower rates of post illness visits and hospitalization and lower hospitalization levels [23–25]. Some studies further explore the influencing factors of health service utilization rates among mobile people, including minority rural migrants. From the persrpective of public service policies themselves, whether health service projects are charged has a complex impact on the coverage, utilization rates, and social costs of health services [3, 26, 27]. In comparison to the abundant literature focusing on the utilization and influencing factors of public health services, there is relatively less research on the impact of health rights accessibility. In particular, some of the related literature has focused on macrolevel assessments of the economic and social impacts of public service investments, especially the potential driving effects of institutional design, value positions, and the supply quality of public services in urban receiving areas on the migration decisions and citizenship levels of rural migrants [13, 28–30]. Only a small number of studies have empirically analyzed the impact of the equalization of public health services on the social integration of rural migrants, suggesting that the utilization of public health services contributes to enhancing the social integration of rural migrants [31]. Whether public medical services can become a crucial means to eliminate group health inequality and income gaps depends critically on who benefits from the public medical service system [32, 33].

What role does the accessibility of health rights play in the urbanization process of rural migrants? This question, which has significant practical implications, has yet to receive a complete and accurate answer. In this context, based on dynamic monitoring survey data from China’s floating population organized by the National Health Commission, this study systematically assesses the impact of the accessibility of health rights on the urban integration of minority rural migrants, starting from health records management, and analyses the underlying mechanisms involved. This study specifically addresses the following three questions. First, can the accessibility of health rights enhance the urban integration of minority rural migrants? Second, if there are certain positive effects, what could be the underlying mechanism? Third, is there heterogeneity in the positive effects of health rights accessibility within minority rural migrants? This study not only contributes to a reassessment of the role of health rights accessibility in improving the living conditions of minority rural migrants but also aids in a deeper understanding of the development process of equalizing public health services for migrants. This study provides factual evidence for further promoting the equalization of public health services for migrants.

## Literature review and theoretical analysis

### Literature review

The “Assimilation Theory,” “Multiculturalism Theory,” and “Segmented Assimilation Theory” are the three most representative theories in the international discourse on immigrant social assimilation [34–36]. The Assimilation Theory categorizes the process of immigrants’ social assimilation into seven dimensions: cultural adaptation, structural assimilation, marital assimilation, identification assimilation, attitudinal acceptance assimilation, behavioral acceptance assimilation, and secular assimilation [37, 38]. With the increase of multi-ethnic, multi-racial, and multi-religious immigrants, the immigrant community in the United States has become more diverse. Different ethnic groups exhibit a distinct “multiculturalism” feature, leading to the gradual formation of the contrasting perspective of “Multiculturalism Theory,” characterized by diversity and inclusivity [39]. Subsequently, researchers found significant differences in the social assimilation status of different immigrant groups in the destination country, especially among the second generation of immigrants. The differences in personal characteristics, cultural backgrounds, and structural positions determine entirely different assimilation outcomes among various immigrant groups, leading to the proposal of the “Segmented Assimilation Theory” [40, 41]. Chinese scholars, building on the international theories of immigrant social assimilation, have proposed the concepts of urban integration and citizenship concerning the situation of rural migrants in China. These concepts are often interchangeably used in the literature. Due to the differences in the socio-economic systems at home and abroad, domestic scholars have a more comprehensive understanding of this issue. They primarily conduct research from three perspectives: the modernization perspective, the socialization perspective, and the perspective of social interaction between rural migrants and urban residents [42–44].

Regarding the factors influencing urban integration, existing studies mainly unfold from macro institutional levels and micro individual levels. First, from an institutional perspective, the household registration system and its derivative systems prevent migrant workers from enjoying the same public services as urban residents, seriously hindering the comprehensive integration of migrant workers into the city [45–48]. Second, from a human capital perspective, migrant workers have a lower level of human capital, especially in terms of individual education and occupational skills, severely restricting their capacity for urban integration [49–51]. Third, from a social capital perspective, inadequate social capital accumulation makes it difficult for migrant workers to break through homogeneous member interaction circles, leading to the reproduction of their marginalized status [16, 36, 52]. Additionally, economic factors such as employment status, income level, housing conditions, occupational achievements, education and training, rural rights, and social security are crucial guarantees for migrant workers to stabilize and integrate into the city [53–58]. Some scholars also study the urban integration of migrant workers from a cultural perspective [59].

Enjoying basic public services is a fundamental right for every citizen, and ensuring that everyone has access to basic public services is also a crucial responsibility of the government. In recent years, the issue of basic public services and its equalization has received extensive attention from the academic community. Previous studies have begun to explore the impact of public services on the citizenship or social integration of migrants. The Tiebout model’s “voting with feet” theory initially focused on the impact of local public service supply on population migration, suggesting that residents express preferences for public services by “voting with their feet” in choosing the direction of movement and residency intentions [28]. Oates’ research on the capitalization of public services and taxes confirmed the existence of the “voting with feet” theory [60]. In fact, due to institutional constraints and insufficient capacity, the level of public services received by rural migrants in China is lower than that of urban local residents [24]. The main reason is that China’s current system of providing basic public services is often tied to household registration, making it difficult for rural migrants to permanently relocate. This poses a significant obstacle for rural migrants, constraining the improvement of their welfare levels. Only a small amount of research has empirically analyzed the impact of equalizing public health services on the social integration of rural migrants, suggesting that the utilization of public health services helps enhance the social integration of rural migrants [31]. The impact of public health services on the settlement intentions of minority mobile populations is greater than that on Han mobile populations [61]. The higher the accessibility and availability of public health rights, the stronger the settlement intentions of minority mobile populations [62].

In summary, research on the relationship between public services and the urban integration of rural migrants is relatively extensive. However, existing studies mostly focus on the overall impact of public services on population mobility. Moreover, these studies often aggregate various types of public services for exploration, with limited specific research dedicated to the accessibility of health rights. Furthermore, the explanatory variables in existing research largely concentrate on the intensity of investment and the supply situation of public health services, with relatively fewer analyses on their underlying mechanisms, presenting a noticeable disparity from our focus on the accessibility of health rights. In China, the health literacy of ethnic minority rural migrant groups is generally low, and their awareness of healthcare is relatively weak, making them a “vulnerable” group in health risk management, more susceptible to diseases. Therefore, the accessibility of health rights for ethnic minority rural migrants in China deserves more attention.

### Theoretical analysis

The core content of public health services is public health. Although most ethnic minority rural migrants contribute to the medical insurance of their household registration location, this medical insurance does not play the expected role for ethnic minority rural migrants who work and live in places other than their registered residence for an extended period. Moreover, reimbursement in a different location is unusually cumbersome and often challenging to obtain through the application process. Health rights are an inclusive and fundamental human right, ensuring the basic guarantee for a dignified life, and everyone has the right to enjoy the highest attainable standard of health fairly and equitably. In China, public health services were originally restricted by the household registration system, serving only the local registered population. In 2009, China initiated the National Basic Public Health Services Project, commencing efforts to equalize public health services for the floating population, gradually providing social security and public health services for rural migrants. Existing studies have found that rural migrants who have already accessed public services such as social security, community health records, health education, and medical insurance have a stronger willingness to migrate [63]. Therefore, the improvement of the accessibility of public health rights in the destination area contributes to enhancing the “sense of attainment” for ethnic minority rural migrants and subsequently promotes their urban integration.

So, what is the mechanism through which the accessibility of health rights affects the urban integration of ethnic minority rural migrants? It is necessary to examine the policies of basic public health service equalization that enhance the accessibility of health rights for ethnic minority rural migrants. Firstly, the core content of public health services is public health, and the original intention of public health is to safeguard the basic human right of health [13]. The improvement of accessibility to health rights in the destination area can enable ethnic minority rural migrants to more widely access, understand, and utilize health information, thereby enriching their health knowledge, enhancing health literacy, fostering good health habits, mitigating health risks, and ultimately improving health levels. At the same time, health, as the foundational component of human capital for ethnic minority rural migrants, not only enriches their urban life, enhances their quality of life and satisfaction, but also, to some extent, determines their ability to integrate into the city. Therefore, there may exist a logical chain of “accessibility of health rights → improvement of health levels → enhancement of urban integration levels.”

Secondly, the improvement in the accessibility of health rights in the destination area can assist ethnic minority rural migrants in acquiring and assimilating scientific and comprehensible medical and health knowledge. This enhancement in health literacy is manifested through the convergence of health habits with urban norms and the cultivation of health-seeking behaviors, thereby positively influencing the urban integration of ethnic minority rural migrants. Specifically, the elevation of health literacy among ethnic minority rural migrants deepens their internal awareness of the consequences of unhealthy behaviors and the benefits of health-promoting behaviors. Combined with external pressures from urban social health norms, this is reflected in the convergence of daily health habits between migrant workers and local residents [13, 64]. Therefore, there may also be a logical chain of “accessibility of health rights → improvement in health habits → enhancement of urban integration levels.” On the other hand, this elevation in health literacy prompts migrant workers to pay more attention to pre-screening, timely consultations, and self-care in disease diagnosis and treatment. This ultimately results in rural migrants being able to choose proactive and correct treatment plans when facing illness [65]. Thus, there may also be a logical chain of “accessibility of health rights → reinforcement of health behaviors → enhancement of urban integration levels.”

## Methods

### Data source

The empirical analysis in this paper is based on data from the 2017 China Migrant Dynamics Survey. The use of this data is primarily based on the following considerations: First, CMDS data is collected by the National Health Commission of China, covering large-scale micro-level survey data in areas with concentrated immigration in 31 provincial-level administrative units in mainland China. It combines authority and large-sample characteristics. Second, the National Health Commission of China is the administrative body responsible for implementing basic public health service projects in China. The CMDS is an important survey activity for the National Health Commission to supervise and track the implementation of public health services for migrants in various regions. The survey targets migrants aged 15 and above who have resided in the destination for more than a month and do not have local (county or city) household registration. It strictly adheres to a stratified, multi-stage, and proportionate sampling method, combining professionalism and scientific characteristics. Third, CMDS2017 data is the latest survey data published by the National Health Commission of China, with strong timeliness. The total sample size of CMDS2017 data is 169,989. Considering questionnaire design and research needs, non-agricultural household migrant populations were excluded, resulting in a final effective sample of 10,727 minority rural migrants.

### Variables

The dependent variable is urban integration. This study comprehensively explored the overall urban integration level of minority rural migrants by constructing a comprehensive urban integration index based on four indicators in two dimensions: city perception and self-perception. In this study, city perception was measured using survey questions such as “I like the city/place where I currently live” and “I pay attention to the city/place where I currently live.” Self-perception is measured using survey questions such as “I am very willing to integrate with local people and become one of them” and “I feel that I am already a local.” The corresponding options included “completely disagree”, “disagree”, “somewhat agree”, and “completely agree” and were assigned values ranging from 1 to 4, respectively.

Additionally, this study considers city perception and self-perception to be equally important for urban integration. City perception emphasizes the life experiences of minority rural migrants upon entering the city, while self-perception underscores their judgment of their own integration level. Given the lack of complete substitutability between the two, this study adopts the average Euclidean distance method proposed by Sarma to construct a comprehensive urban integration index for minority rural migrants [66]. The main idea of this method is to consider multiple dimensions as a point in multidimensional space, with this point corresponding to the urban integration level achieved by minority rural migrants. The calculation formula is as follows:

1 $$ {CI}_{1}=\sqrt{{\sum }_{i=1}^{2}{{d}_{i}}^{2}}/\sqrt{2}, {CI}_{2}=1-\sqrt{{\sum }_{i=1}^{2}(1-{{d}_{i}}^{2})}/\sqrt{2}, CI=({CI}_{1}+{CI}_{2})/2$$

2 $$ {SI}_{1}=\sqrt{{\sum }_{i=3}^{4}{{d}_{i}}^{2}}/\sqrt{2 }, {SI}_{2}=1-\sqrt{{\sum }_{i=3}^{4}(1-{{d}_{i}}^{2})}/\sqrt{2}, SI=({SI}_{1}+{SI}_{2})/2$$

3 $$ {I}_{1}=\sqrt{({CI}^{2}+{SI}^{2})}/\sqrt{2}, {I}_{2}=1-\sqrt{(1-{CI}^{2})+(1-{SI}^{2})}/\sqrt{2 }, I=({I}_{1}+{I}_{2})/2$$

Among them, $$ {CI}_{1}$$, $$ {CI}_{2}$$, and $$ CI$$ represent the distance from the actual point to the worst point, the reverse distance to the optimal point, and the average distance on the city perception level, which is the urban integration index on the city perception level, covering the first two subindices. $$ {SI}_{1}$$, $$ {SI}_{2}$$, and $$ SI$$ represent the corresponding values on the self-perception level, including the last two subindices. $$ {I}_{1}$$, $$ {I}_{2}$$, and $$ I$$ represent the actual distance from the point to the worst point, the reverse distance to the optimal point, and the average distance, respectively, considering both city perception and self-perception. $$ I$$ is the overall index of urban integration for minority rural migrants.

The explanatory variable is the accessibility of health rights. The explanatory variable is the accessibility of health rights. This study measures the accessibility of health rights for minority rural migrants using the “whether a health record is established” variable. Currently, local governments in China primarily rely on health records to carry out various public health service projects for migrants, including health education, infectious disease prevention and control, and healthcare management for mobile pregnant women and children. The establishment of health records directly affects the type and content of health services that minority rural migrants can access. This variable is operationalized in the questionnaire as follows: “Has the local area established a health record for you?” The respondents could choose from options such as “Yes, already established,” “Not established, never heard of it,” “Not established, but heard of it,” or “Unclear.” In this study, “Yes, already established” is assigned a value of 1, while the other situations are assigned a value of 0. Within the sample scope, 2,545 minority rural migrants, accounting for 23.73%, had established health records.

Considering the possibility of endogeneity issues, this study conducts tests using the instrumental variable method. The accessibility of health rights for minority rural migrants in their destination cities mainly depends on the policy efforts of local governments to provide public health services. In 2013, the National Health Commission of China issued the “Work Plan for Pilot Equalization of Basic Public Health and Family Planning Services for Migrants.” Currently, 44 cities with a relatively concentrated floating population in 31 provincial-level administrative units in mainland China have implemented the equalization of basic public health and family planning services for migrants (see Table A1). In these 44 key cities, rural migrants can enjoy the same public health service rights as local residents, significantly enhancing the accessibility and level of health rights for minority rural migrants. However, whether minority rural migrants’ destination cities are selected as key cities generally does not directly impact their urban integration. Therefore, we select “Key Cities for Equalization of Public Health Services” as the instrumental variable for the accessibility of health rights for minority rural migrants.

This study considers health level, health habits, and health behaviors as mechanisms through which the accessibility of health rights influences the urban integration of minority rural migrants. This study considers health level, health habits, and health behaviors as mechanisms through which the accessibility of health rights influences the urban integration of minority rural migrants. Health habits were measured using the questionnaire item “My hygiene habits are no different from those of local residents,” with options such as “Completely disagree,” “Disagree,” “Basically agree,” and “Completely agree,” assigned values of 1, 2, 3, and 4, respectively. Health behaviors were measured using the questionnaire item “Did you seek timely medical attention when you had cold symptoms in the past year?” with the option “Yes” assigned a value of 1 and “No” assigned a value of 0.

This study controls for various potential confounding factors that may simultaneously affect the accessibility of health rights for minority rural migrants and their urban integration, including gender, age, marital status, education level, mobility range, duration of local residence, income level, employment status, and family size. Additionally, considering policy variations across different regions (provincial or municipal administrative units), this study controls for regional effects in the form of dummy variables. The meanings and descriptive statistics of each variable are shown in Table 1.

**Table 1:** Descriptive statistics of the variables

Variable	Value	Mean	Std.
Urban integration	Calculated from the average Euclidean distance method	0.478	0.278
Health rights accessibility	Is a resident health file established? 1 = Established, 0 = Other	0.237	0.425
Gender	1 = Male, 0 = Female	0.497	0.500
Age	Respondent’s age in 2017 (years)	34.007	10.606
Marriage	1 = Married, 0 = Unmarried	0.748	0.434
Education	1 = High school and above, 0 = Below high school	0.255	0.436
Migration range	1 = Inter-provincial mobility, 2 = Intra-provincial inter-city, 1 = Intra-city inter-county	1.797	0.732
Residence time	Duration of residence in the city (years)	5.279	5.789
Income	Logged family per capita income (Yuan)	7.436	0.762
Occupation	Respondent’s current employment status: 1 = Self-employed (Employers or self-employed workers), 0 = Other (Employee or other employment status)	0.285	0.452
Family size	Number of family members living together	3.181	1.340
Rural homestead	Whether or not there is a rural homestead in your hometown: 1 = Yes, 0 = No	0.589	0.492
Health level	Self-reported health. 1 = unable to care for oneself, 2 = Unhealthy, but self-sufficient, 3 = Basically healthy, 4 = Healthy	3.771	0.498
Healthy habits	My personal hygiene habits are not different from those of local citizens. 1 = completely disagree, 2 = disagree, 3 = generally agree, 4 = completely agree	2.905	0.776
Health behavior	Whether or not they sought medical attention when they had a cold or flu in the last year: 1 = Yes, 0 = No	0.259	0.438

### Empirical strategies

First, this study constructs the following regression equation to assess the impact of the accessibility of health rights on the urban integration of minority rural migrants:

4 $$ {Urban\_integration}_{i}=\alpha +\beta {Health\_rights}_{i}+\gamma {W}_{i}+{\epsilon }_{i}$$

where $$ {Urban\_integration}_{i}$$ represents the level of urban integration for minority rural migrants. $$ {Health\_rights}_{i}$$ represents the accessibility of health rights, $$ {W}_{i}$$ includes a series of control variables, $$ \alpha $$, $$ \beta $$, and $$ \gamma $$ are the parameters to be estimated, and $$ {\epsilon }_{i}$$ is the random disturbance term.

Second, it is worth noting that the above baseline regression may still have potential endogeneity. The level of urban integration for minority rural migrants may directly influence their expectations of staying in the city, thereby affecting their enthusiasm and initiative in utilizing health rights upon migration, leading to reverse causation. Additionally, in the regression process, the omission of important variables is possible, and omitted variables can result in biased regression results. The instrumental variable method is a common approach for addressing endogeneity issues and requires constructing a regression equation for the accessibility of health rights and its instrumental variables before estimating Eq. (4):

5 $$ {Health\_rights}_{i}=\theta +\phi {Z}_{i}+\omega {W}_{i}+{u}_{i} $$

In Eq. (5), $$ {Z}_{i}$$ is the instrumental variable; $$ \theta $$, $$ \phi $$, and $$ \omega $$ are the parameters to be estimated; and $$ {u}_{i}$$ is the error term. This study employs a 2SLS model to correct for potential endogeneity in the baseline regression process. However, considering that the potential endogenous variable, the accessibility of health rights, is a binary variable, the 2SLS model somewhat neglects its categorical nature, not fully utilizing the information, and causes some loss of estimation efficiency. Therefore, this study introduces the conditional mixed process (CMP), a method capable of fitting a series of multiple equations and multilevel and conditional recursive mixed-process estimators to re-estimate the instrumental variables within a unified CMP framework [67]. CMP, based on seemingly unrelated regression, constructs a recursive system of equations using the maximum likelihood estimation method, requiring simultaneous estimation of Eqs. (4) and (5). By utilizing the arctangent correlation coefficient (atanhrho) of the error terms of the two equations, it can be determined whether the accessibility of health rights variable is an endogenous variable. If atanhrho is significantly different from 0, the CMP estimation results are superior to the baseline regression results.

Third, there was a change in the analytical perspective. If, after adding as many reasonable control variables as possible in the analysis, the coefficient of the core explanatory variable remains stable, this implies that even if there are omitted variables, it is difficult to “overturn” the core conclusions of this study [68]. This study uses the method proposed by Oster to test potential omitted variables and their impact on the regression results [69], i.e., when there are some unobservable omitted variables in the regression model, obtaining an approximate consistent estimate of the effect of the accessibility of health rights on the urban integration of minority rural migrants by calculating the estimator ${\beta }^{\text{*}}$:

6 $$ {\beta }^{*}\approx \stackrel{\sim}{\beta }-\delta \left({\beta }^{0}-\stackrel{\sim}{\beta }\right)\times \left({R}_{max}-\stackrel{\sim}{R}\right)/\left(\stackrel{\sim}{R}-{R}^{0}\right)$$

where $$ {\beta }^{*}$$ represents the impact of the accessibility of health rights on the urban integration of minority rural migrants and $$ {\beta }^{0}$$ and $$ {R}^{0}$$, respectively denote the parameter estimate and goodness of fit of health rights accessibility when adding constrained control variables. $$ \stackrel{\sim}{\beta }$$ and $$ \stackrel{\sim}{R}$$ represent the parameter estimate and goodness of fit of health rights accessibility when all observable variables are added as control variables. $$ \delta $$ represents the ratio of explanatory power between observable variables and unobservable variables for the urban integration of minority rural migrants. $$ {R}_{\text{m}\text{a}\text{x}}$$ represents the maximum goodness of fit of the regression equation when all omitted variables can be included in the model. Following Oster’s suggestion [69], we assume that $$ {R}_{\text{m}\text{a}\text{x}}$$ is 1.2, 1.4, 1.6, 1.8, and 2 times the current goodness of fit of the regression equation. A value of $$ \delta $$ greater than 1 when $$ \beta $$=0 indicates that the omitted variables will not change the impact of the explanatory variable on the dependent variable.

## Empirical analysis

### Baseline regression results

Table 2 reports the baseline regression results for the impact of health rights accessibility on the urban integration of minority rural migrants. To validate the robustness of the regression results, we adopt a stepwise regression approach: (1) controlling only for core explanatory variables, (2) adding control variables, (3) adding provincial dummy variables, and (4) replacing provincial dummy variables with municipal dummy variables. Table 2 shows that, controlling for only core explanatory variables, adding control variables and provincial dummy variables, or adding municipal dummy variables, the impact of health rights accessibility on the urban integration of minority rural migrants is significantly positive at the 1% level, indicating the robustness of the estimation results. Column (4) shows that compared to establishing health records, establishing health records will increase the urban integration level of minority rural migrants by 6%. The above results suggest that improving the accessibility of health rights can effectively promote the urban integration of minority rural migrants.

Additionally, the control variable results in Column (4) show that the older the respondent is, the greater the urban integration level of minority rural migrants. Marital status and education level have a significantly positive impact on the urban integration of minority rural migrants, indicating that married individuals and those with a high school education or above have a greater level of urban integration. Both the migration range and duration have a significantly positive impact on the urban integration of minority rural migrants, suggesting that those with a smaller migration range and longer local residence have a greater level of urban integration. Compared to minority rural migrants without a rural homestead, those with a rural homestead have a lower level of urban integration. Overall, the estimated results are consistent with the findings of the literature [70–73].

**Table 2:** Baseline Regression Results: The overall impact of health rights accessibility on the urban integration of minority rural migrants

Variable	(1)	(2)	(3)	(4)
Health rights accessibility	0.091***	0.078***	0.060***	0.060***
	(0.007)	(0.006)	(0.007)	(0.007)
Gender		0.004	0.003	0.006
		(0.005)	(0.005)	(0.005)
Age		0.002***	0.002***	0.002***
		(0.000)	(0.000)	(0.000)
Marriage		0.015**	0.014*	0.018**
		(0.008)	(0.007)	(0.007)
Education		0.034***	0.028***	0.030***
		(0.006)	(0.006)	(0.006)
Migration range		0.050***	0.032***	0.020***
		(0.004)	(0.005)	(0.005)
Residence time		0.006***	0.006***	0.006***
		(0.001)	(0.001)	(0.001)
Income		-0.007*	0.000	0.007
		(0.004)	(0.004)	(0.005)
Occupation		0.029***	0.018***	0.007
		(0.006)	(0.006)	(0.006)
Family size		0.003	0.003	0.002
		(0.003)	(0.003)	(0.003)
Rural homestead		-0.049***	-0.027***	-0.021***
		(0.005)	(0.006)	(0.006)
Regional effect	NO	NO	YES	YES
Constant	0.457***	0.314***	0.325***	0.375***
	(0.003)	(0.038)	(0.043)	(0.051)
R^2^	0.019	0.093	0.133	0.194
Observations	10,727	10,727	10,727	10,727

Note: Robust standard errors in parentheses. Provincial regional effects are shown in column (3), and city regional effects are shown in column (4) of Table 3. *** *p* < 0.01, ** *p* < 0.05, * *p* < 0.1

### Endogeneity

#### Instrumental variable checks

Despite controlling for potential influencing variables on health rights accessibility and urban integration in the baseline regression analysis, the empirical analysis may still face potential endogeneity issues such as reverse causation and omitted variable bias. Therefore, we further analyze the results using the 2SLS model, and Table 3 reports the estimation results of the 2SLS model. From the estimation results of the first stage of the 2SLS model in Column (1), it can be observed that the instrumental variable has a significant positive impact on health rights accessibility, indicating that the instrumental variable meets the relevance condition. The endogeneity test parameter of health rights accessibility is significant at the 5% level, suggesting that health rights accessibility is indeed an endogenous variable and that the estimation results of the 2SLS model are more reliable than those of the OLS model. According to the estimation results of the second stage of the 2SLS model in Column (2), an increase in health rights accessibility significantly promotes the urban integration of minority rural migrants. This result is consistent with the previous estimation results, demonstrating the robust positive impact of health rights accessibility on the urban integration of minority rural migrants.

Additionally, due to the binary nature of the endogenous variable health rights accessibility, the use of the 2SLS model somewhat reduces the effectiveness of the estimation results. Therefore, this study further utilizes the CMP method for estimation. From the regression results of the first stage of the CMP method in Column (3), it can be observed that the instrumental variable has a significant positive impact on health rights accessibility, confirming that the instrumental variable satisfies the relevance requirement. The regression results of the second stage of the CMP method in Column (4) show that health rights accessibility has a significantly positive impact on the urban integration of minority rural migrants, with a coefficient of 0.071. Compared to the coefficient in Column (2), this indicates a loss of effectiveness in the estimation of the 2SLS model. According to the endogeneity test results, the atanhrho_12 value is significant at the 10% level, rejecting the null hypothesis that health rights accessibility is an exogenous variable. After endogeneity treatment, health rights accessibility has a significantly positive impact on the urban integration of minority rural migrants, and these results are highly consistent with the estimation results of the OLS model and the 2SLS model. Therefore, the positive impact of health rights accessibility on the urban integration of minority rural migrants is robust.

**Table 3:** Regression results with the 2SLS model and CMP

Variable	2SLS			CMP	
	(1)	(2)		(3)	(4)
Health rights accessibility		0.732*			0.071***
		(0.428)			(0.014)
Instrumental variable	0.071**			0.305**	
	(0.034)			(0.145)	
Constant	0.756***	-0.131		-1.343***	0.368***
	(0.053)	(0.328)		(0.287)	(0.051)
Control variables	YES	YES		YES	YES
City effect	YES	YES		YES	YES
Observations	10,727	10,727		10,727	10,727

Note: Robust standard errors in parentheses. *** *p* < 0.01, ** *p* < 0.05, * *p* < 0.1

#### Omitted variable checks

Table 4 reports the results of the omitted variable test based on the Oster method. When $$ \beta $$=0, whether $$ {R}_{max}$$ is taken as 1.2, 1.4, or 1.6, 1.8, or 2 times, all $$ \delta $$ values are greater than 1. This indicates that the coefficient of the impact of health rights accessibility on the urban integration of minority rural migrants remains stable. Therefore, even in the presence of omitted variables, the judgment of the relationship between health rights accessibility and urban integration in this study remains robust, indicating that health rights accessibility significantly enhances the urban integration of minority rural migrants.

**Table 4:** Omitted variable check results

Variable	Standard of judgment	$$ {R}_{max}$$					Pass the test
		1.2$\stackrel{\sim}{R}$	1.4$\stackrel{\sim}{R}$	1.6$\stackrel{\sim}{R}$	1.8$\stackrel{\sim}{R}$	2.0$\stackrel{\sim}{R}$	
Health rights accessibility	> 1	5.068	2.735	1.873	1.424	1.149	Yes

### Robustness

To further validate the reliability of the empirical results, we conducted robustness tests through adjustments to both the explanatory and dependent variables, as shown in Table 5. First, we conducted robustness tests using three variables highly correlated with the explanatory variable. The first variable measured health rights accessibility through the questionnaire item “In the past year, have you received health education on disease prevention in your current village/residence?” Those who had received at least one health education item were assigned a value of 1, while those who had not received any health education were assigned a value of 0. The second variable measured health rights accessibility through the questionnaire item “In the past year, have you received free follow-up assessments, health check-ups, etc., for hypertension or type II diabetes provided by the local community health service center/station/township hospital?” Those who answered “Yes” were assigned a value of 1, and those who answered “No” were assigned a value of 0. The results of the regression with adjusted explanatory variables are presented in columns (1) and (2), which show that even with different indicators of health rights accessibility, the conclusions in Table 2 remain robust.

Additionally, we adjusted the assignment method for the dependent variable. First, we used an equal-weight weighting method instead of the average Euclidean distance method to reverse the urban integration level of minority rural migrants. The results of the regression are presented in column (3). Second, we directly replaced the dependent variable with one measure of urban integration, i.e., “I am willing to integrate with local people and become one of them.” The results of the regression are presented in column (4). The estimated results show that health rights accessibility remains highly significant, with a significantly positive coefficient, indicating that health rights accessibility still significantly enhances the urban integration level of minority rural migrants. This further confirms the robustness of the core conclusion.

**Table 5:** Results of the robustness check

Variable	(1)	(2)	(3)	(4)
Health rights accessibility	0.029***	0.041**	0.039***	0.100***
	(0.006)	(0.019)	(0.004)	(0.016)
Constant	0.418***	0.424***	0.702***	3.520***
	(0.045)	(0.050)	(0.039)	(0.101)
Control variables	YES	YES	YES	YES
City effect	YES	YES	YES	YES
R^2^	0.189	0.188	0.205	0.145
Observations	10,727	10,727	10,727	10,727

Note: Robust standard errors in parentheses. *** *p* < 0.01, ** *p* < 0.05, * *p* < 0.1. Health education specifically covers nine aspects: occupational disease prevention and control, prevention and control of sexually transmitted diseases/HIV/AIDS, reproductive health and contraception, tuberculosis prevention and control, smoking control, mental health, chronic disease prevention and control, maternal and child health/eugenics, and self-rescue in emergencies related to public health

### Heterogeneous effects

In the previous sections, we concluded that the accessibility of health rights contributes to the enhanced urban integration of minority rural migrants. However, it is crucial to note that this is an overall average effect at the sample level, without considering the heterogeneity of this impact. To obtain more nuanced research findings, we grouped the data based on generation, mobility range, and illness experience and conducted a heterogeneous analysis. The detailed estimation results are presented in Table 6.

Columns (1) and (2) report the differences between different generations. Overall, whether they belong to the older or the newer generation of minority rural migrants, the accessibility of health rights significantly and positively influences their urban integration. Additionally, the size of the impact, as observed through the Suest method based on seemingly unrelated models, suggested no significant differences between the generations. This is mainly because high-quality public service resources are a shared pursuit for both older and newer generations of minority rural migrants and a primary goal in their rural-to-urban migration [13, 74, 75].

Columns (3) and (4) report the differences between different mobility ranges. The results show that, whether migrants are within a province or across provinces, the accessibility of health rights significantly and positively influences urban integration. However, the Suest test results indicate that the promoting effect of health rights accessibility on urban integration is more significant for those migrating within the province. This is mainly because, compared to inter-provincial migrants, minority rural migrants within a province may find it easier to adapt, accept, and utilize the high-quality public service resources of their target urban areas [12, 76].

Columns (5) and (6) report the differences between different illness experiences. The results show that, regardless of whether there is a history of illness, the accessibility of health rights significantly and positively influences urban integration. However, the Suest test results indicate that the promoting effect of health rights accessibility on urban integration is more significant for minority rural migrants with a history of illness. This is mainly because, compared to those without an illness history, minority rural migrants with such a history are more likely to enhance their willingness to integrate into the city due to the accessibility of health rights [14, 77, 78].

**Table 6:** Heterogeneous effects of the availability of health rights

Variable	After 1980	Before 1980		Inter-provincial	Within-province		Illness history	Non illness history
	(1)	(2)		(3)	(4)		(5)	(6)
Health rights accessibility	0.063***	0.055***		0.036***	0.071***		0.071***	0.051***
	(0.008)	(0.013)		(0.010)	(0.009)		(0.010)	(0.009)
Constant	0.455***	0.122*		0.438***	0.368***		0.121**	0.321***
	(0.060)	(0.064)		(0.063)	(0.053)		(0.059)	(0.066)
Control variables	YES	YES		YES	YES		YES	YES
City effect	YES	YES		YES	YES		YES	YES
R^2^	0.190	0.229		0.245	0.154		0.230	0.192
Suest test	15.480			25.690***			26.230***	
Observations	6,949	3,491		4,178	6,549		4,991	5,736

Note: Robust standard errors in parentheses. *** *p* < 0.01, ** *p* < 0.05, * *p* < 0.1

### Analysis of mediating Effect

The above analysis indicates that the accessibility of health rights significantly enhances the urban integration of minority rural migrants. Therefore, how can we explain the underlying mechanisms? To examine the mechanisms through which the accessibility of health rights influences the urban integration of minority rural migrants, this study, combining the aforementioned analysis with the availability of data, employs the Karlson et al. proposed Karlson-Holm-Breen (KHB) decomposition method [79]. This method assesses the promoting effects of health rights accessibility through three aspects: health level, health habits, and health behaviors. The aim is to comprehensively reveal the relationships between health rights accessibility and the urban integration of minority rural migrants.

Table 7 reports the estimation results based on the KHB method. According to the results in column (1), the indirect effect of health level is significantly positive at the 1% level, indicating that health rights accessibility indirectly promotes the urban integration of minority rural migrants by enhancing their health levels. Similarly, as evident from columns (2) and (3), the indirect effects of health habits and health behaviors are also significantly positive at the 1% level. This finding implies that health rights accessibility indirectly propels urban integration by improving health habits and strengthening health behaviors. Further analysis revealed that the percentages of the total effect of health rights accessibility on the urban integration of minority rural migrants mediated by health habits, health level, and health behaviors were 1.98%, 3.08%, and 0.79%, respectively. Hence, health rights accessibility not only directly promotes the urban integration of minority rural migrants but also indirectly enhances their urban integration by improving health levels, modifying health habits, and reinforcing health behaviors. Notably, health habits play a more significant role in the indirect effect.

**Table 7:** Regression results with mediators

Variable	Health level	Healthy habits	Health behavior
	(1)	(2)	(3)
Total effect	0.257***	0.260***	0.257***
	(0.026)	(0.026)	(0.026)
Direct effect	0.252***	0.252***	0.254***
	(0.026)	(0.026)	(0.026)
Indirect effect	0.005***	0.008**	0.002**
	(0.002)	(0.004)	(0.001)
Control variables	YES	YES	YES
City effect	YES	YES	YES
Observations	10,727	10,727	10,727

Note: Robust standard errors in parentheses. *** *p* < 0.01, ** *p* < 0.05, * *p* < 0.1

## Discussion

The right to health is a broadly inclusive fundamental human right, serving as a fundamental guarantee for humans to live with dignity, and everyone has the right to enjoy the highest health standards that are fair and accessible. In China, the health literacy of rural migrant populations is generally low, and health awareness is relatively weak, making them a “vulnerable” group in terms of health risk management and more susceptible to diseases. Therefore, the quality of public health services for rural migrants should receive increased attention. Providing urban public health services to rural migrants is a crucial pathway for the government to safeguard the health rights and interests of rural migrants. In this regard, the “Healthy China 2030” planning outline emphasizes the need to carry out the equalization of basic public health and family planning services for the floating population. It comprehensively includes long-term mobile populations residing for six months or more in public health service projects, ensuring equal access to public health services. Research has focused mostly on the current status of health service utilization and its influencing factors among rural migrants, with a noticeable lack of discussion and assessment regarding the health rights accessibility of minority rural migrants [80, 81]. The health rights and interests of ethnic minority rural migrants in China face certain practical challenges. According to CMDS2017 data, firstly, ethnic minority rural migrants face various health risks. About 35.48% of ethnic minority rural migrants are engaged in commerce and production, exposing them to greater mental stress and higher levels of hazardous substances or equipment. Moreover, lacking awareness of health security, 72.85% of ethnic minority rural migrants have not established health records, and 86.18% have not participated in medical insurance in their destination areas. Secondly, factors such as ethnic culture, lifestyle, medical habits, and language norms may hinder ethnic minority rural migrants from accessing public health benefits and improving the accessibility of public health rights in their destination areas. Zhao et al. have also highlighted the cultural barriers to health equity faced by rural migrants across dialectal regions [12]. Hence, taking a novel perspective on health rights accessibility, this study employs instrumental variable methods, omitted variable tests, and mediation models to systematically examine the overall impact, heterogeneity, and mechanisms of health rights accessibility on the urban integration of minority rural migrants, ultimately obtaining robust and credible research conclusions.

The distribution pattern of ethnic populations in China, characterized by large-scale dispersion, small-scale clustering, and intertwined coexistence, continues to deepen, displaying new features of significant mobility and extensive integration. Meanwhile, the healthcare service demands of rural ethnic minority migrants in China are substantial, with frequent interruptions in health services. Consequently, health management faces certain practical challenges. Using CMDS 2017 data, this study evaluates the impact and mechanisms of health rights accessibility on the urban integration of minority rural migrants. One of our most significant findings is that improving the level of health rights accessibility significantly enhances the urban integration of minority rural migrants. This positive effect persists even after controlling for potential endogeneity and performing a series of robustness tests. The accessibility of health rights at a destination not only relates to the health status and subjective welfare level of minority rural migrants but also has potential implications for their competitiveness in urban integration. Due to the risks of frequent mobility, vulnerability to mobile employment, difficulties in social integration, and continuity of social security, minority rural migrants are prone to health risks. Our work not only contributes to reevaluating the value of public health services in improving the living conditions of rural migrants but also deepens our understanding of the development process of equalizing public health services for migrants. This provides empirical evidence for further promoting the equalization of public health services for minority rural migrants.

Meanwhile, we found that the urban integration level of male minority rural migrants is significantly greater than that of female minority rural migrants. This finding is consistent with the conclusions of most related studies, primarily because male minority rural migrants typically possess strong adaptability and competitiveness, allowing them to adapt more quickly to urban life and work rhythms and integrate better into urban society [55, 82]. Compared to unmarried minority rural migrants, married individuals have a greater urban integration level. This is because married minority rural migrants, who have a stable family, prioritize maintaining family life in urban settings, resulting in a greater level of urban integration than unmarried individuals [71, 72]. The higher the educational level of minority rural migrants is, the greater their urban integration level. Similar results have been found in related studies, indicating that minority rural migrants with higher educational levels have relatively higher employment levels and incomes in urban areas, and their economic capability for urban integration is continuously strengthening [83]. Compared to minority rural migrants moving within the province, those moving across provinces exhibit significantly lower levels of urban integration. The greater the distance of mobility is, the lower the level of urban integration, possibly due to significant differences in language, lifestyle, and cultural traditions between the destination city and the household registration location, thereby increasing the difficulty of urban integration for minority rural migrants [84]. The longer minority rural migrants stay at a destination, the greater their level of urban integration, indirectly reflecting the positive impact of the stability of work and life on psychological integration. At the same time, the longer minority rural migrants stay at the destination, the closer their social distance is to the locals, making them more eager to become citizens [85]. Minority rural migrants with rural residential land have a lower level of urban integration. This clearly indicates that the influence of rural residential land factors on the urban integration of rural migrants cannot be ignored. The greater the expected compensatory income from rural residential land is, the lower the willingness of minority rural migrants to reside and integrate into the city [73, 86].

These heterogeneous results indicate that the impact of health equity accessibility on the urban integration of minority rural migrants varies with respect to mobility scope and illness experience. Specifically, the enhancing effect of health equity accessibility on urban integration is more significant for minority rural migrants moving within the province and those with illness experience. This is primarily because cross-provincial mobility implies being away from one’s hometowns and relatives and facing more challenges in adapting to and integrating into urban society. Minority rural migrants moving within the province have advantages in terms of culture, language, and social relationships, and they also enjoy more abundant public health service resources. Hence, they can better integrate into urban society [12, 76]. Additionally, health equity accessibility has a more substantial facilitating effect on urban integration for minority rural migrants with illness experience. This is mainly because only when minority rural migrants encounter diseases and genuinely experience the medical benefits brought about by local health equity accessibility does health equity accessibility positively influence their urban integration [77, 78].

Additionally, this study revealed that health status, health habits, and health behaviors are the mechanisms through which health equity accessibility influences the urban integration of minority rural migrants. The improvement of health equity accessibility can help minority rural migrants enhance their health levels, improve health habits, and strengthen health behaviors, thereby promoting their urban integration. Related research indicates that good health status may enrich the urban life of minority rural migrants, enhance their quality of life and satisfaction, and potentially elevate their level of urban integration [14]. Moreover, our study suggested that a significant improvement in health equity accessibility enhances the health literacy of minority rural migrants, helping them cultivate good health habits, avoid health risks, and ultimately increase their health levels. Previous research has shown that providing public health services can make minority rural migrants feel cared for and supported in the city, reduce their psychological distance from the city, enhance their sense of urban belonging and identity, and facilitate the social integration of minority rural migrants with local residents [13, 87]. Additionally, our research indicates that enhancing health equity accessibility at destinations can assist minority rural migrants in acquiring and absorbing scientific and understandable medical and health knowledge and promoting the formation of health-seeking behaviors, thereby enhancing their urban integration.

Finally, it should be pointed out that, despite utilizing nationwide large-sample survey data organized by the Chinese National Health Commission and employing various methods for empirical analysis in this study, there are still limitations. For rural migrants, there is currently no long-term tracking survey data available. Analyzing with cross-sectional data is challenging on the one hand because it is difficult to capture the dynamic processes of health equity accessibility and urban integration among minority rural migrants. On the other hand, it is also not possible to use fixed-effects models to control for the influence of individual differences that do not change over time.

## Conclusion

Attaining health rights is an integral part of people’s aspiration for a better life. The accessibility of health equity at a destination not only affects the subjective welfare of minority rural migrants but also may impact their health conditions and urban integration competitiveness. Based on CMDS 2017 data, we systematically evaluated the impact of health equity accessibility on the urban integration of minority rural migrants from the perspective of health records, analyzing the underlying mechanisms involved. We found that improving health equity accessibility can enhance the urban integration of minority rural migrants, and this enhancement effect is more pronounced among those who move within the province and those with illness experience. Moreover, the influence of health equity accessibility on the urban integration of minority rural migrants not only has a direct effect but also indirectly enhances their urban integration through health habits, health levels, and health behaviors.

The conclusions of this study have important policy implications. In advancing the process of rural migrant urbanization, efforts should focus on enhancing the health equity accessibility of minority rural migrants, continuously improving their ability to obtain health rights at their destination. Specifically, first, efforts should be made to enhance the quality and efficiency of public health education. Tailor personalized health education plans and programs based on the actual conditions and needs of minority rural migrants are needed to ensure that educational content can be effectively absorbed and applied. Simultaneously, education methods and approaches should be optimized, enhancing the specificity and effectiveness of education. The diversity and flexibility of educational methods should be emphasized to meet the needs of different groups of minority rural migrants. Second, the publicity and popularization of health knowledge among minority rural migrants should be strengthened. Various channels and methods, such as community bulletin boards, radio, television, and the internet, are utilized to convey health knowledge to minority rural migrants, increasing their health awareness and self-care capabilities. Additionally, health lectures, training sessions, and other activities should be organized, and professional doctors or health education experts should be invited to provide on-site guidance for minority rural migrants, helping them better understand and master health knowledge.

### Electronic supplementary material

Below is the link to the electronic supplementary material.

Supplementary Material 1

## Data Availability

The datasets used in the present study are publicly available from the China Migration Population Service Center [https://www.chinaldrk.org.cn].
