# Supplementary material for: Effect of health rights accessibility on the urban integration of minority rural migrants in China: a cross-sectional study
Source: BMC Public Health. 2024 Mar 11;24:761. doi: 10.1186/s12889-024-18294-3 (PMC10929153; doi:10.1186/s12889-024-18294-3)
Supplement: Supplementary file 1 — Supplementary Material 1 [file 12889_2024_18294_MOESM1_ESM.docx]

# Appendix

**Table A1** List of key cities for the equalization of basic public health services for the floating population in China

| No. | Province | City | No. | Province | City |
| --- | --- | --- | --- | --- | --- |
| 1 | Beijing | Chaoyang | 23 | Fujian | Xiamen |
| 2 |  | Fengtai | 24 |  | Quanzhou |
| 3 | Tianjin | Binhai | 25 | Jiangxi | Nanchang |
| 4 |  | Jinan | 26 | Shandong | Qingdao |
| 5 | Hebei | Shijiazhuang | 27 | Henan | Zhengzhou |
| 6 | Shanxi | Taiyuan | 28 | Hubei | Wuhan |
| 7 | Inner Mongolia | Baotou | 29 | Hunan | Changsha |
| 8 | Liaoning | Dalian | 30 | Guangdong | Shenzhen |
| 9 | Jilin | Changchun | 31 |  | Zhongshan |
| 10 | Heilongjiang | Harbin | 32 | Guangxi | Guilin |
| 11 | Shanghai | Minhang | 33 | Hainan | Sanya |
| 12 |  | Yangpu | 34 | Chongqing | Yubei |
| 13 |  | Songjiang | 35 | Sichuan | Chengdu |
| 14 |  | Baoshan | 36 | Guizhou | Guiyang |
| 15 | Jiangsu | Nanjing | 37 | Yunnan | Yuxi |
| 16 |  | Suzhou | 38 | Tibet | Lhasa |
| 17 |  | Wuxi | 39 | Shaanxi | Xi 'an |
| 18 | Zhejiang | Hangzhou | 40 |  | Xianyang |
| 19 |  | Ningbo | 41 | Gansu | Lanzhou |
| 20 |  | Jiaxing | 42 | Qinghai | Xining |
| 21 |  | Shaoxing | 43 | Ningxia | Yinchuan |
| 22 | Anhui | Hefei | 44 | Xinjiang | Karamay |
